# Integrative proteomics and pharmacogenomics analysis of methylphenidate treatment response

**DOI:** 10.1038/s41398-019-0649-5

**Published:** 2019-11-18

**Authors:** Bruna S. da Silva, Douglas T. Leffa, Walter O. Beys-da-Silva, Iraci L. S. Torres, Diego L. Rovaris, Marcelo M. Victor, Luis A. Rohde, Nina R. Mota, Carla de Oliveira, Markus Berger, John R. Yates, Renuka Sabnis, Ramón Díaz Peña, Alexandre Rosa Campos, Eugenio H. Grevet, Lucelia Santi, Claiton H. D. Bau, Verônica Contini

**Affiliations:** 1 Department of Genetics, Institute of Biosciences, Universidade Federal do Rio Grande do Sul, Porto Alegre, Brazil; 2 ADHD Outpatient Program, Adult Division, Hospital de Clínicas de Porto Alegre, Porto Alegre, Brazil; 3 Postgraduate Program in Medical Sciences, School of Medicine, Universidade Federal do Rio Grande do Sul, Porto Alegre, Brazil; 4 Faculty of Pharmacy, Universidade Federal do Rio Grande do Sul, Porto Alegre, Brazil; 5 Center of Experimental Research, Hospital de Clínicas de Porto Alegre, Porto Alegre, Brazil; 6 Pharmacology Department, Institute of Basic Health Sciences, Universidade Federal do Rio Grande do Sul, Porto Alegre, Brazil; 7 Department of Psychiatry, School of Medicine, Universidade Federal do Rio Grande do Sul, Porto Alegre, Brazil; 8 Department of Human Genetics, Donders Institute for Brain, Cognition and Behaviour, Radboud University Medical Center, Nijmegen, The Netherlands; 9 Department of Chemical Physiology, Scripps Research, La Jolla, CA USA; 10 Proteomics Facility, Sanford-Burnham-Prebys Medical Discovery Institute, 10901N. Torrey Pines Road, La Jolla, CA 92037 USA; 11 Postgraduate Program in Biotechnology, Universidade do Vale do Taquari - Univates, Lajeado, Brazil

**Keywords:** Pharmacogenomics, ADHD

## Abstract

Transcriptomics and candidate gene/protein expression studies have indicated several biological processes modulated by methylphenidate (MPH), widely used in attention-deficit/hyperactivity disorder (ADHD) treatment. However, the lack of a differential proteomic profiling of MPH treatment limits the understanding of the most relevant mechanisms by which MPH exerts its pharmacological effects at the molecular level. Therefore, our aim is to investigate the MPH-induced proteomic alterations using an experimental design integrated with a pharmacogenomic analysis in a translational perspective. Proteomic analysis was performed using the cortices of Wistar-Kyoto rats, which were treated by gavage with MPH (2 mg/kg) or saline for two weeks (*n* = 6/group). After functional enrichment analysis of the differentially expressed proteins (DEP) in rats, the significant biological pathways were tested for association with MPH response in adults with ADHD (*n* = 189) using genome-wide data. Following MPH treatment in rats, 98 DEPs were found (*P* < 0.05 and FC < −1.0 or > 1.0). The functional enrichment analysis of the DEPs revealed 18 significant biological pathways (gene-sets) modulated by MPH, including some with recognized biological plausibility, such as those related to synaptic transmission. The pharmacogenomic analysis in the clinical sample evaluating these pathways revealed nominal associations for gene-sets related to neurotransmitter release and GABA transmission. Our results, which integrate proteomics and pharmacogenomics, revealed putative molecular effects of MPH on several biological processes, including oxidative stress, cellular respiration, and metabolism, and extended the results involving synaptic transmission pathways to a clinical sample. These findings shed light on the molecular signatures of MPH effects and possible biological sources of treatment response variability.

## Introduction

Methylphenidate (MPH) is a widely used stimulant for pharmacological treatment of attention-deficit/hyperactivity disorder (ADHD). Its safety and efficacy for ADHD treatment in both adults and children are supported by meta-analyses; however, a considerable proportion of patients do not present a satisfactory response^1–3^. The main direct targets of MPH are the dopamine and norepinephrine transporters (DAT and NET, respectively). In addition, the interactions of MPH with other proteins of these neurotransmission systems, such as dopaminergic, adrenergic, and AMPA receptors^4–6^, as well as the modulation of signaling of other neurotransmitters, including serotonin, glutamate, and GABA^7–9^, are suggested to be involved in its pharmacological actions. Moreover, several other pathways such as those involved in neuronal plasticity, energy metabolism, cell differentiation, circadian rhythm, and ubiquitin-dependent protein degradation are also affected by MPH, as evidenced by transcriptomics and candidate gene/protein expression studies^10–17^. Although these studies used different methodologies, including in vivo and in vitro treatments, the overall evidence indicates that MPH actions involve a complex biological scenario, but the most relevant mechanisms for treatment response are still unclear.

Neuroimaging studies can complement the elucidation of MPH actions by pointing out the most relevant regions involved, and they suggest that MPH attenuates some ADHD-related structure alterations (e.g., reduced volumes of gray matter), mainly in basal ganglia and anterior cingulate cortex regions^18,19^. Functional studies reported that MPH mitigates ADHD dysfunctions in the prefrontal cortex, anterior cingulate cortex, and striatum. Of note, MPH treatment influences ventral frontostriatal functional connectivity without tasks^20^, as well as activates prefrontal cortex, basal ganglia, and cerebellum during tasks^21,22^. This set of evidence suggests that MPH effects involve mainly frontal and striatal regions, and a comprehensive investigation of the changes induced by MPH in such regions can provide a better understanding of its pharmacological effects.

Proteomic technology is a promising tool to investigate the molecular basis of ADHD, and can also help to clarify the existing gaps regarding MPH actions. This high-throughput hypothesis-free technique provides broad coverage of the brain profile, and the differential global protein expression under different conditions can shed light on specific-state biological underpinnings^23^. For example, proteomic molecular signatures of psychostimulants, including cocaine and methamphetamine, have suggested alterations in metabolism, oxidative stress, degenerative process, mitochondrial dysfunction, synaptic proteins, and plasticity^24–26^. Even though previous studies have explored MPH-induced effects in the expression of particular proteins, we are not aware of investigations using a hypothesis-free approach to characterize the proteomic profile according to MPH treatment. In addition, pharmacogenomic studies of MPH are scarce, especially in adults, precluding further insights on the molecular basis of its effects.

**Fig. 1:**
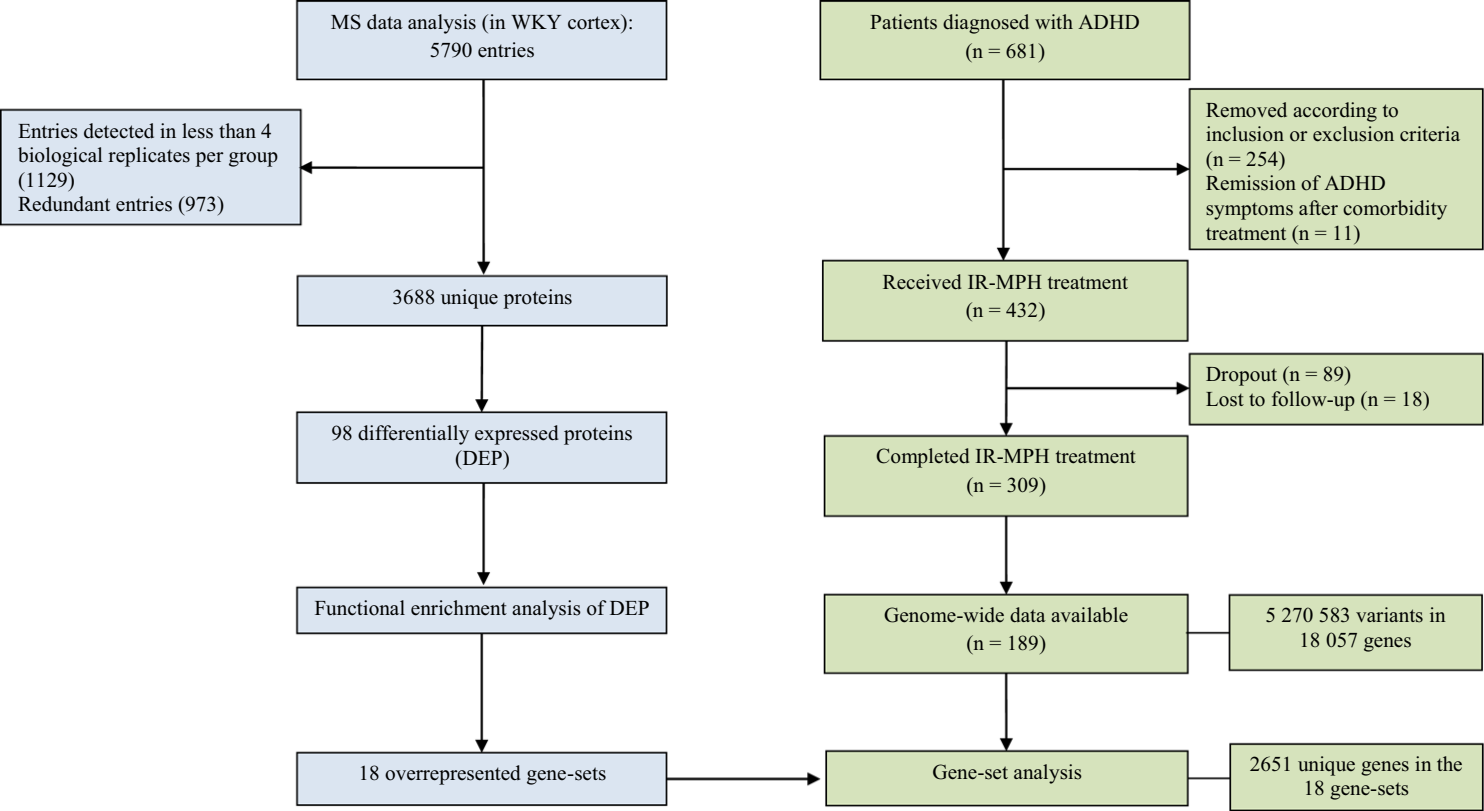
Flowchart of data analysis for proteomics (left) and pharmacogenomics (right). ADHD attention-deficit/hyperactivity disorder, IR-MPH immediate-release methylphenidate, MS mass spectrometry, WKY Wistar-Kyoto.

Therefore, this study aims to identify the MPH-induced alterations in the proteome of cortex tissue of Wistar-Kyoto (WKY) rats. Functional enrichment of the differentially expressed proteins (DEPs) into biological pathways was performed to provide an overview of the systems affected by MPH treatment. Moreover, from a translational perspective, the proteomic results were used to generate hypotheses to be tested for association with MPH treatment response in a clinical sample of adults with ADHD, using genome-wide data. The rationale of our study design and the details on the proteomics and pharmacogenomics analyses are shown in Fig. 1. This integrative approach, in addition to providing general insights for biological pathways modulated by MPH treatment, points out which of these pathways may also influence MPH treatment response variability.

## Materials and methods

### Differential proteomics of rats treated with MPH

#### Animals

WKY rats, males, and adults (90–120 days) weighing 260–320 g were used in this experiment. The rats were housed in 49 × 34 × 16 cm polypropylene cages (maximum of 4 per cage) at a temperature of 22 °C ± 2 °C on a 12-h light/dark cycle (lights on at 7 a.m.). Animals had ad libitum access to food and water. A period of 2 weeks for the stabilization and acclimation to the environment was provided to the animals prior to the initiation of the interventions. All the experimental design of this study was approved by the Institutional Committee for Animal Care and Use (GPPG-HCPA protocol no. 20160346) and performed in accordance with the Guide for the Care and Use of Laboratory Animals 8th edition (2011). The maintenance of the animals followed the law 11.794 (Brazil), which establishes procedures for the scientific use of animals. Vigorous attempts were made to minimize animal suffering, and decrease external sources of pain and discomfort, as well as to use the minimum number of animals required to produce reliable scientific data.

#### Drugs

Immediate-release methylphenidate (IR-MPH; Ritalin, Novartis, Brazil) was dissolved in 0.9% saline solution before the administration to the rats by gavage at a dose of 2 mg/kg. This dose results in plasma concentrations similar to what is considered clinically effective^27–30^.

#### Experimental design

WKY rats were randomly assigned to control (saline-treated) or MPH-treated groups. Each group was composed by six animals (biological replicates), which is in the range that usually provides relevant results among proteomic animal studies. The animals received IR-MPH or 0.9% saline solutions both by gavage twice a day for 14 days. Treatment was performed at the same time and by the same researcher (7 a.m. and 7 p.m. daily). Approximately 12 h after the last IR-MPH dose, animals were killed by decapitation and the brains were instantly removed for dissection. The cortices were used for this experiment, and they were immediately processed and prepared for protein extraction (the flowchart of the experimental design is provided in Supplementary Fig. 1).

#### Sample preparation and protein extraction for mass spectrometry

After dissection, the cerebral cortex tissues were individually homogenized and sonicated on cold HEPES/sucrose buffer containing protease and phosphatase inhibitors. The proteins were extracted using methanol and chloroform and stored at −80 °C until further processing. Protein extracts were resuspended with 8 M urea 50 mM ammonium bicarbonate, and protein concentration was determined using a bicinchoninic acid (BCA) protein assay (Thermo Scientific). Proteins were then digested, acidified with formic acid and subsequently desalted using AssayMap C18 cartridges (Agilent) mounted on an Agilent AssayMap BRAVO liquid handling system. More detailed procedures on the preparation of proteins prior to liquid chromatography-tandem mass spectrometry (LC-MS/MS) analysis are described in the Supplementary Methods.

#### Mass spectrometry

Dried samples were reconstituted with 2% acetonitrile, 0.1% formic acid and analyzed by LC-MS/MS using a Proxeon EASY 1200 nanoLC system (Thermo Fisher Scientific) coupled to an Orbitrap Fusion Lumos mass spectrometer (Thermo Fisher Scientific). Peptide separation was performed on a 25 cm column with 75 µm inner diameter packed in-house with ethylene bridged hybrid (BEH) C18 1.7 µm resin (Waters) in a 180-min gradient of 6–45% of solvent B (80% acetonitrile, 0.1% formic acid) at a flow rate of 310 nL/min. The mass spectrometer was operated in positive data-dependent acquisition mode. MS1 spectra were measured with a resolution of 120,000, an automatic gain control (AGC) target of 1e6, a maximum injection time of 100 ms and a mass range from 350 to 1500 m/z. The instrument was set to run in top speed mode with 3 s cycles for the survey and the MS/MS scans. After a survey scan, tandem MS was performed on the most abundant precursors exhibiting a charge state from 2 to 8 of greater than 5e4 intensity by isolating them in the quadrupole at 0.8 Th. High-energy collision dissociation (HCD) fragmentation was applied with 30% collision energy, and resulting fragments were detected using the turbo scan rate in the ion trap. The AGC target for MS/MS was set to 1e4, and the maximum injection time limited to 15 ms. The dynamic exclusion was set to 20 s with a 10 ppm mass tolerance around the precursor and its isotopes. MS data are available via ProteomeXchange with identifier PXD015473.

#### Proteomics data analysis

The MaxQuant software version 1.5.5.1 was used to analyze the mass spectra. MS/MS spectra were searched against the *Rattus norvegicus* Uniprot protein sequence database (version January 2016) and the common Repository of Adventitious Proteins (cRAP) sequences (commonly known protein contaminants) from Global Proteome Machine database. Evidence table output from MaxQuant was used for label-free protein quantitative analysis. First, calculated peptide intensities were log base 2 transformed and normalized across samples to account for systematic errors. Following normalization, all non-razor peptide sequences were removed from the list, and protein-level quantification and testing for differential abundance were performed using MSstats Bioconductor package^31^. The model decomposes log-intensities into the effects of biological replicates, peptides, and statistical interactions. Specific settings used in MaxQuant and further details on the normalization procedure can be found in Supplementary Methods. For all these procedures performed in each biological replicate, no identification of group allocation was provided during the experiment.

Since MaxQuant cluster unresolved protein redundancy into protein groups, the entry with a higher number of peptides matched to the protein was selected to be included in the analysis. Proteins were excluded if not detected in at least 4 out of 6 animals. Proteins presenting a log base 2 transformed fold-change (Log2FC) higher than 1.0 (upregulated with MPH) or lower than −1.0 (downregulated with MPH) plus a nominal *P*-value < 0.05 were considered as significantly differentially expressed between the MPH-treated and saline-treated groups. The consideration of a cutoff for effect sizes (Log2FC) in addition to the *P*-value helps to reduce the probability of false-positive results without eliminating true-positive identifications that occur when applying traditional methods of multiple testing correction in quantitative proteomics^32^.

#### Functional enrichment analysis of DEPs

The DEPs (as gene names; Table 1) were analyzed using FUMA web application v1.3.3c^33^ to obtain information on the biological context in which these proteins are inserted. FUMA is an integrative web-based platform that provides pathway enrichment results by performing hypergeometric tests to evaluate whether genes of interest are overrepresented in any of the pre-defined sets from different categories. The categories of interest for this study in terms of biological mechanisms include Gene Ontology (GO) Biological Process, Kyoto Encyclopedia of Genes and Genomes (KEGG), and Reactome. False Discovery Rate (FDR) correction taking into account the total number of gene-sets in each category was used to determine the significantly overrepresented gene-sets, with an adjusted *P*-value cutoff of 0.05. The minimum number of overlapped genes was set as 2. Additional tools (DAVID and Enrichr) were used as a complementary approach to compare the results and outline the most consistently enriched pathways among the different software. DAVID test the significance of gene-term enrichment with a modified Fisher’s exact test (EASE score; set as 0.05), which compares the percentage of input genes that comprises a particular pathway to this proportion in the whole genome^34^. Enrichr retrieves *P*-values based on Fisher’s exact test, but it precomputes a background expected rank for each term in each gene-set category^35^.

**Table 1:** Differentially expressed proteins with MPH treatment.

Accession number	Protein names	Gene names	Log2FC (MPH/Saline)	P-value
Q3ZAU5^a^	DDHD domain-containing 1	*Ddhd1*	−11.053	0.0100
A0A0G2K6R0^a^	Trinucleotide repeat-containing 6B	*Tnrc6b*	−10.937	0.0125
Q63540^a^	Ataxin-1	*Atxn1*	−10.600	0.0125
F1MAR6^a^	Proline dehydrogenase	*Prodh1*	−10.133	0.0100
P10818	Cytochrome c oxidase subunit 6A1, mitochondrial	*Cox6a1*	−3.693	0.0001
Q6LED0	Histone H3.1	*Hist1h3b*	−2.876	0.0052
D4A5X7	Ganglioside-induced differentiation-associated-protein 1	*Gdap1*	−2.108	0.0040
Q04940	Neurogranin	*Nrgn*	2.082	0.0185
D4ADS4	Microsomal glutathione S-transferase 3	*Mgst3*	−2.048	0.0088
P14056	Serine/threonine-protein kinase A-Raf	*Araf*	−1.982	0.0063
B4F7E8	Niban-like protein 1	*Fam129b*	−1.864	0.0025
F1MA59	Collagen type IV alpha 1 chain	*Col4a1*	−1.776	0.0038
A0A0H2UHZ1	Aquaporin-4	*Aqp4*	−1.734	0.0147
Q62717	Calcium-dependent secretion activator 1	*Cadps*	−1.732	0.0032
M0R3V7	Sodium/calcium exchanger 1	*Slc8a1*	−1.731	0.0157
Q0VGK0	Gamma-aminobutyric acid receptor-associated protein-like 1	*Gabarapl1*	−1.713	0.0146
A0A0U1RRQ1	NADH:ubiquinone oxidoreductase subunit A1	*Ndufa1*	−1.656	0.0108
F1M779	Clathrin heavy chain;Clathrin heavy chain 1	*Cltc*	−1.653	0.0182
P30835	ATP-dependent 6-phosphofructokinase, liver type	*Pfkl*	−1.651	0.0350
Q812E9	Neuronal membrane glycoprotein M6-a	*Gpm6a*	−1.630	0.0464
P23978	Sodium- and chloride-dependent GABA transporter 1	*Slc6a1*	−1.615	0.0417
Q6MFX9	Myelin-oligodendrocyte glycoprotein	*Mog*	−1.608	0.0176
A0A0G2K4L6	Similar to TSC22 domain family protein 2	*Tsc22d2*	1.574	0.0084
Q6P9Y4	ADP/ATP translocase 1	*Slc25a4*	−1.540	0.0410
F1LUT4	Phospholipid-transporting ATPase	*Atp8a1*	−1.501	0.0410
A0A0G2JZK7	Sodium/calcium exchanger 2	*Slc8a2*	−1.495	0.0384
P11505	Plasma membrane calcium-transporting ATPase 1	*Atp2b1*	−1.481	0.0310
A0A0G2K757	Dolichyl-diphosphooligosaccharide–protein glycosyltransferase subunit 2	*Rpn2*	−1.441	0.0222
F1M110	Glycylpeptide N-tetradecanoyltransferase	*Nmt2*	−1.437	0.0032
M0RDI5	Mitochondrial calcium uniporter	*Mcu*	−1.410	0.0361
A0A0G2JWE3	Protein IWS1 homolog	*Iws1*	−1.393	0.0126
Q07984	Translocon-associated protein subunit delta	*Ssr4*	−1.388	0.0274
P31647	Sodium- and chloride-dependent GABA transporter 3	*Slc6a11*	−1.375	0.0489
A0A0G2K6E2	Thioredoxin reductase 2, mitochondrial	*Txnrd2*	−1.368	0.0052
F1LQB2	Structural maintenance of chromosomes protein	*Smc3*	−1.368	0.0408
Q4V7D9	Acid sphingomyelinase-like phosphodiesterase	*Smpdl3b*	−1.363	0.0065
Q63584	Transmembrane emp24 domain-containing protein 10	*Tmed10*	−1.354	0.0125
Q63564	Synaptic vesicle glycoprotein 2B	*Sv2b*	−1.316	0.0382
Q68FW7	Threonine–tRNA ligase, mitochondrial	*Tars2*	−1.305	0.0136
P00762	Anionic trypsin-1	*Prss1*	1.302	0.0019
D4A106	WD repeat domain 3	*Wdr3*	−1.298	0.0035
A0A0G2K9V6	Threonine–tRNA ligase, cytoplasmic	*Tars*	−1.298	0.0301
D3ZEI4	Hepatocyte cell adhesion molecule	*Hepacam*	−1.289	0.0177
A0A0G2JSU4	N-myc downstream regulated gene 2, isoform CRA_b	*Ndrg2*	−1.285	0.0310
P18088	Glutamate decarboxylase 1	*Gad1*	−1.278	0.0145
B0BN81	40S ribosomal protein S5	*Rps5*	−1.251	0.0461
P06685	Sodium/potassium-transporting ATPase subunit alpha-1	*Atp1a1*	−1.234	0.0333
B0BNG3	Lectin, mannose-binding 2	*Lman2*	−1.231	0.0040
O35165	Golgi SNAP receptor complex member 2	*Gosr2*	−1.223	0.0148
Q02563	Synaptic vesicle glycoprotein 2A	*Sv2a*	−1.220	0.0440
D4ACM1	Elongator complex protein 3	*Elp3*	−1.211	0.0303
B2GUV7	Eukaryotic translation initiation factor 5B	*Eif5b*	−1.208	0.0005
Q566R4	Leucine repeat adapter protein 2	*Fam89b*	1.206	0.0116
D3ZYT2	Mitochondrial ribosomal protein S5	*Mrps5*	1.205	0.0349
Q6AY18	SAR1 gene homolog A (S. cerevisiae), isoform CRA_b	*Sar1a*	−1.190	0.0174
B1PLB1	CD34 antigen	*Cd34*	1.189	0.0259
F1LQG0	Huntingtin-associated protein 1	*Hap1*	1.187	0.0075
O89035	Mitochondrial dicarboxylate carrier	*Slc25a10*	−1.187	0.0434
Q4V898	RNA-binding motif protein, X chromosome	*Rbmx*	−1.184	0.0027
F1LS72	Ubiquitin-like modifier-activating enzyme 2	*Uba2*	−1.183	0.0182
P16975	SPARC	*Sparc*	1.170	0.0073
D3ZE85	DOMON domain-containing protein FRRS1L	*Frrs1l*	−1.170	0.0019
B4F774	Ganglioside-induced differentiation-associated protein 1-like 1	*Gdap1l1*	−1.169	0.0383
D3ZZ21	NADH dehydrogenase (Ubiquinone) 1 beta subcomplex, 6	*Ndufb6*	−1.163	0.0072
D4ABI7	Very-long-chain (3 R)-3-hydroxyacyl-CoA dehydratase	*Hacd3*	−1.150	0.0362
G3V728	4-nitrophenylphosphatase domain and non-neuronal SNAP25-like protein homolog 1 (C. elegans), isoform CRA_b	*Nipsnap1*	−1.145	0.0012
D3ZQD3	Oxoglutarate dehydrogenase-like	*Ogdhl*	−1.133	0.0207
F1M471	EPM2A-interacting protein 1	*Epm2aip1*	−1.130	0.0173
D4A8N2	Ferredoxin 2	*Fdx2*	1.112	0.0170
A1L1M0	cAMP-dependent protein kinase catalytic subunit alpha	*Prkaca*	−1.117	0.0358
G3V746	Glutamate receptor ionotropic, NMDA 2B	*Grin2b*	−1.106	0.0364
Q5XI78	2-oxoglutarate dehydrogenase, mitochondrial	*Ogdh*	−1.102	0.0280
D3ZZN3	Acetyl-coenzyme A synthetase	*Acss1*	−1.101	0.0071
F1LMR7	Dipeptidyl aminopeptidase-like protein 6	*Dpp6*	−1.101	0.0146
D4AD70	60S ribosomal protein L38	*Rpl38*	−1.097	0.0003
D3ZIS5	Cytochrome c oxidase assembly factor COX19	*Cox19*	1.091	0.0161
Q3SWS9	Janus kinase and microtubule-interacting protein 1	*Jakmip1*	1.073	0.0111
Q5PPG6	Nucleosome assembly protein 1-like 5	*Nap1l5*	1.070	0.0081
B4F7A9	Casein kinase 2 alpha 2	*Csnk2a2*	−1.054	0.0031
P84100	60S ribosomal protein L19	*Rpl19*	−1.052	0.0373
Q4KM74	Vesicle-trafficking protein SEC22b	*Sec22b*	−1.048	0.0462
Q5XIJ4	Protein FAM210A	*Fam210a*	−1.035	0.0177
A0A0G2K4T7	General transcription factor II-I	*Gtf2i*	−1.031	0.0020
Q641Y2	NADH dehydrogenase [ubiquinone] iron-sulfur protein 2, mitochondrial	*Ndufs2*	−1.029	0.0243
P62332	ADP-ribosylation factor 6	*Arf6*	−1.025	0.0081
A0A0G2K490	TRAF2 and NCK-interacting kinase	*Tnik*	−1.021	0.0419
B2GV54	Neutral cholesterol ester hydrolase 1	*Nceh1*	−1.019	0.0211
Q9JIX3	Bis(5-adenosyl)-triphosphatase	*Fhit*	−1.019	0.0008
A0A0H2UHV9	Coatomer subunit gamma-2	*Copg2*	−1.015	0.0028
P00388	NADPH–cytochrome P450 reductase	*Por*	−1.011	0.0321
Q5XI38	Lymphocyte cytosolic protein 1	*Lcp1*	−1.010	0.0488
D4A4F9	RCG20461	*Stum*	−1.007	0.0223
A0A0G2K9J0	Tetratricopeptide repeat, ankyrin repeat and coiled-coil-containing 2	*Tanc2*	−1.006	0.0333
D4A3D9	Serine/threonine kinase 32C	*Stk32c*	−1.005	0.0134
D4A193	Receptor expression-enhancing protein	*Reep1*	−1.004	0.0222
A2RRU1	Glycogen [starch] synthase, muscle	*Gys1*	−1.003	0.0374
Q4QQV3	Protein FAM162A	*Fam162a*	−1.001	0.0055
Q6PST4	Atlastin-1	*Atl1*	−1.001	0.0484

*MPH* methylphenidate, *Log2FC* log base 2 transformed fold-change

^a^Not detected by the proteomic technique in any biological replicate from the MPH-treated group. The Log2FC and *P*-value are imputed values. The former was calculated as the average of Log2 protein intensity divided by 3.3, while imputed *P*-value was calculated as 0.05 divided by the number of replicates the specific protein was detected across replicates of the detected condition. The effects of MPH treatment on the expression of proteins are indicated by negative (downregulated) or positive (upregulated) Log2FC values

### Gene-set analysis of MPH-treated adults with ADHD

In order to evaluate which of the gene-sets generated from functional enrichment analysis of the DEPs of the cortex of rats would also be involved with MPH treatment response in humans, a gene-set analysis using genome-wide data was performed for a clinical sample of adults with ADHD treated with IR-MPH. Considering the molecular targets of MPH are comparable in healthy or pathological conditions, this approach intends to translate the proteomic findings of the molecular effects of MPH in brain tissue of rats to a clinical perspective focused on the treatment response variability. Therefore, it is expected to reveal not only the molecular signatures of MPH but also in which pathways the genetic variability would be relevant for treatment response to MPH.

#### Sample

This sample comprised 189 individuals of European descent who were ascertained from the ADHD Outpatient Program in the adult division at *Hospital de Clínicas de Porto Alegre* (HCPA), Brazil. Diagnostic procedures for ADHD followed DSM-IV criteria^36^, and other lifetime psychiatric comorbidities were assessed using the Structured Clinical Interview for DSM-IV Axis I Disorders (SCID-I)^37^. The inclusion criteria were: (a) being white Brazilian of European descent; (b) aged 18 years or older; (c) fulfillment of the Diagnostic and Statistical Manual of Mental Disorders, Fourth Edition, (DSM-IV) diagnostic criteria for ADHD;^36^ and (d) eligibility to IR-MPH treatment. The exclusion criteria were: (a) contraindication for IR-MPH use; (b) current stimulant treatment; (c) evidence of a clinically significant neurological disease that might affect cognition (for example, delirium, dementia, epilepsy, head trauma and multiple sclerosis), (d) current or past history of psychosis, and (e) estimated intelligence quotient score lower than 70. Detailed characteristics of the sample are shown in Supplementary Table 1. More detailed information on the sample has been described elsewhere^38,39^. This study was carried out in accordance with the Declaration of Helsinki, and all participants signed an informed consent form previously approved by the institutional review board of the hospital (No. 00000921).

### Treatment and outcome measure

The pharmacological intervention followed the Brazilian guidelines for a more extensive study on clinical predictors of treatment response to IR-MPH^40^. Patients with unstable psychiatric comorbidities were treated with appropriate medications before initiation of treatment with IR-MPH, and they were later re-evaluated to confirm the ADHD diagnosis. After stabilization, they received IR-MPH standard initial daily dose of 10 mg, with doses increasing weekly until symptom control or occurrence of limiting adverse effects. At least 30 days of treatment were required to define the endpoint. The evaluation of treatment response was assessed considering the continuous variable of percentage reduction in severity scores of ADHD symptoms of the Portuguese version of the Swanson, Nolan and Pelham Rating Scale, version 4 (SNAP-IV) from baseline to endpoint. The quantitative measure of treatment response (percentage reduction in symptom severity) rather than a stratification of the sample into groups of responders versus non-responders was chosen since it provides higher statistical power to detect associations.

#### Genotyping

DNA was extracted from peripheral blood. The individuals were genotyped on the Infinium PsychArray-24 BeadChip (Illumina, San Diego, CA, USA). This microarray includes 265,000 SNPs from the Infinium Core-24 BeadChip, 245 000 markers from the Infinium Exome-24 BeadChip, and 50,000 additional markers previously associated with common psychiatric disorders. The data were processed at the Stanley Center for Psychiatric Research, Broad Institute of MIT and Harvard (Cambridge, MA, USA). Quality control (QC), principal components analysis, and genotype imputation procedures were implemented using the default values on RICOPILI following the PGC pipeline (https://sites.google.com/a/broadinstitute.org/ricopili/home). The European population of the 1000 Genomes Project Phase 1 was used as the reference panel. Postimputation QC was performed using the following settings: info score > 0.6, minor allele frequency > 1%, call rate > 98% and Hardy-Weinberg equilibrium test with *P* > 1e-06. The resulting dataset after QC consisted of 5,270,583 SNPs in 18,057 genes for 189 individuals.

#### Gene-set analysis

MAGMA v1.07b was used to perform the gene-set analysis following the basic steps of the software guidelines that include: annotation, gene analysis, and gene-set analysis^41^. For SNP annotation to genes, gene locations for build 37 (hg19) were used, setting a 2 kb upstream and 1 kb downstream window. For gene analysis, the principal components regression model was used. MAGMA’s gene analysis uses multiple regression to address linkage disequilibrium between genome-wide markers and to detect multi-marker effects^41^. Potential confounding variables were included in the analysis when associated with the outcome. Based on this, concomitant use of medication and baseline severity scores (assessed by the clinical global impression—severity (CGI-S) scale) were included as covariates, as well as the ten first principal components to control for population stratification. In the gene-set analysis, each of the 18 enriched pathways generated from the DEPs in rats was tested for association with MPH treatment response in the clinical sample (measured by the percentage change of symptoms between the baseline and the endpoint). The competitive testing uses the results from gene analysis to evaluate whether the combined effect of genes comprising the gene-sets is larger than all other genes in the genome (not included in the gene-sets) in relation to the outcome. FDR was applied for multiple testing correction considering all the gene-sets tested.

## Results

### Differential proteomics of rats treated with MPH

#### Identification of DEPs

According to our pre-defined criteria of Log2FC and *P*-value, 98 proteins showed differential expression with MPH treatment (86 proteins were considered downregulated, and 12 upregulated; Table 1). Among them, four proteins (DDHD1, TNRC6B, ATXN1, and PRODH1) were exclusively detected in the saline-treated group, while no protein was expressed only in the MPH-treated group. Few of these proteins showed substantial differential expression between groups with Log2FC higher than + 2.0 (NRGN) or lower than −2.0 (COX6A1, HIST1H3B, GDAP1, and MGST3), and most of them showed subtler changes with Log2FC of ± 1.5.

#### Functional enrichment analysis of DEPs

**Fig. 2:**
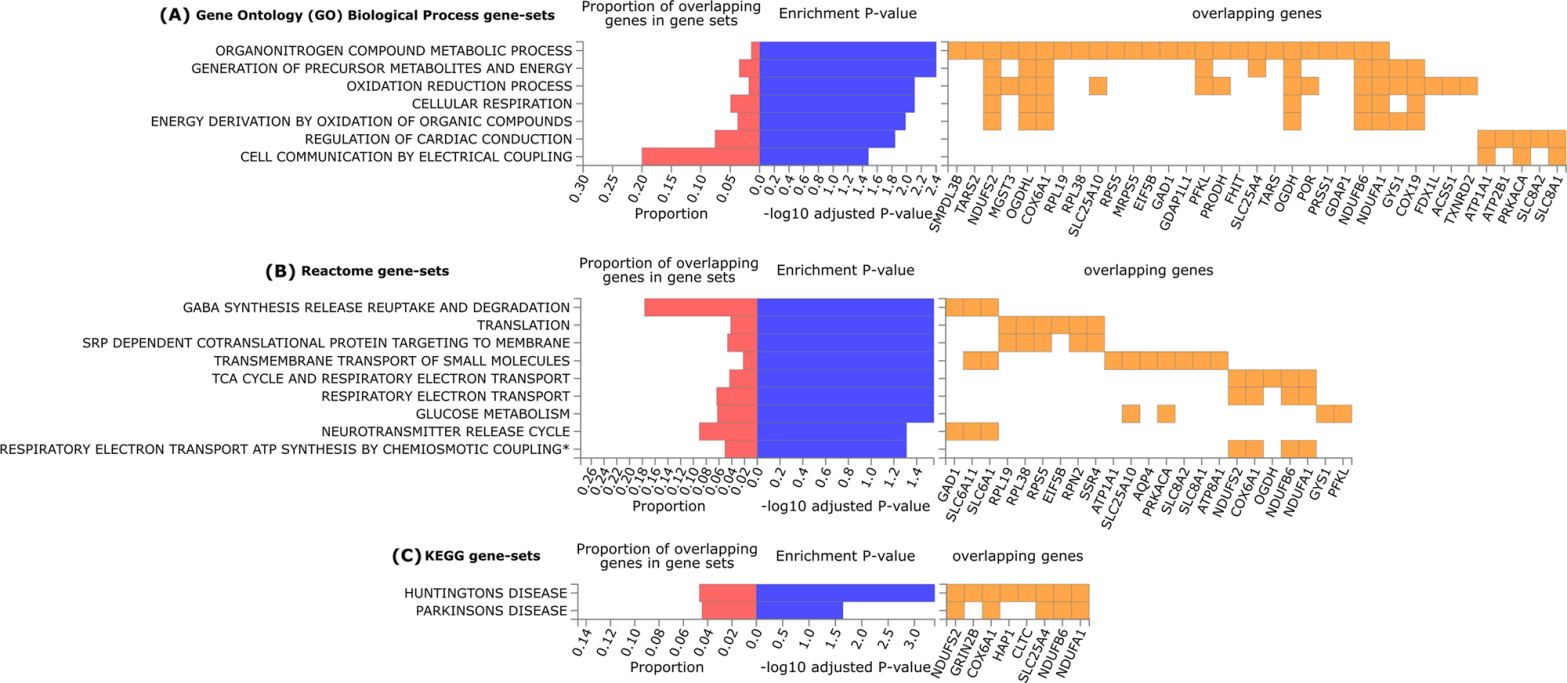
FUMA results of the overrepresented gene-sets retrieved from the functional enrichment analysis of the 97 differentially expressed proteins with MPH treatment in the cortex of Wistar-Kyoto rats. The blue bars represent the enrichment *P*-value (-log10 adjusted) after FDR correction considering the number of gene-sets tested per category. The red bars indicate the proportion of overlapping inputted genes according to the size (number of genes) of each gene-set. The orange squares show the inputted genes that are part of the enriched gene-sets. (**a**) Significantly enriched gene-sets from the Gene Ontology (GO)—Biological Process category. (**b**) Significantly enriched gene-sets from the Reactome category. (**c**) Significantly enriched gene-sets from the Kyoto Encyclopedia of Genes and Genomes (KEGG) category. *Respiratory electron transport ATP synthesis by chemiosmotic coupling and heat production by uncoupling proteins.

The enrichment analysis of the DEPs (entered as gene names) into functional categories using FUMA resulted in seven significantly overrepresented pathways (gene-sets) from GO Biological process (Fig. 2a), nine from Reactome (Fig. 2b) and two from KEGG (Fig. 2c) categories after FDR correction. This analysis was based on 97 genes, since the *Stum* gene (Uniprot protein accession code D4A4F9) was not recognized by FUMA, even under alternative names/codes. The complementary analyses using DAVID and Enrichr software can be found in Supplementary Tables 2 and 3, respectively. We observed differences in the association values retrieved by these tools. For example, although several pathways were overrepresented in DAVID with EASE cutoff of 0.05, only Reactome Muscle Contraction remains significant after FDR correction (Supplementary Table 2), while Enrichr retrieves several significantly enriched pathways from Reactome and KEGG categories after FDR correction, but none from the GO Biological Process category (Supplementary Table 3). Despite the differences in the statistical significance, in general, there is a relatively high concordance of the overrepresented pathways among the tools used, and we will focus the discussion on the principal results from FUMA, taking into account the most consistent findings among the three tools.

### Gene-set analysis of MPH-treated adults with ADHD

All 18 gene-sets retrieved by FUMA from the three categories that were significantly overrepresented from the proteomic analysis were tested for association with IR-MPH treatment response in a clinical sample of adults with ADHD (Table 2). Gene-set analysis for the percentage change in severity scores of ADHD after IR-MPH treatment resulted in nominal associations for two gene-sets from the Reactome category: the GABA synthesis release reuptake and degradation, containing 17 genes (*P* = 0.013), and Neurotransmitter release cycle, containing 32 genes (*P* = 0.008). However, these associations did not remain significant after FDR correction (*P*_corr_ = 0.117 and *P*_corr_ = 0.117, respectively). In the analysis stratifying the sample by sex, the nominal associations observed for the whole sample remain only in men (*P* = 0.043 and *P*_corr_ = 0.387; *P* = 0.009 and *P*_corr_ = 0.162, respectively; Supplementary Table 4). No associations were found for the gene-sets from the GO Biological Process and KEGG categories.

**Table 2:** Competitive gene-set analysis of the percentage change in symptoms of ADHD according to SNAP-IV scale after treatment with IR-MPH in adults with ADHD.

Gene-set	Number of genes	Beta (SE)	*P*-value
*Gene ontology (GO)—biological process*			
Organonitrogen compound metabolic process	1685	0.007 (0.021)	0.365
Generation of precursor metabolites and energy	272	−0.005 (0.047)	0.538
Oxidation reduction process	840	0.025 (0.029)	0.191
Cellular respiration	134	0.025 (0.064)	0.348
Energy derivation by oxidation of organic compounds	202	−0.028 (0.054)	0.694
Regulation of cardiac conduction	63	−0.163 (0.105)	0.939
Cell communication by electrical coupling	15	0.309 (0.201)	0.063
*Reactome*			
GABA synthesis release reuptake and degradation	17	0.455 (0.205)	**0.013**^**a**^
Translation	133	0.031 (0.067)	0.322
SRP dependent cotranslational protein targeting to membrane	100	0.032 (0.075)	0.335
Transmembrane transport of small molecules	391	0.009 (0.044)	0.429
TCA cycle and respiratory electron transport	111	0.018 (0.071)	0.401
Respiratory electron transport	62	0.029 (0.092)	0.378
Glucose metabolism	59	0.066 (0.103)	0.261
Neurotransmitter release cycle	32	0.375 (0.155)	**0.008**^**b**^
Respiratory electron transport ATP synthesis by chemiosmotic coupling and heat production by uncoupling proteins	78	−0.007 (0.082)	0.534
*Kyoto Encyclopedia of Genes and Genomes (KEGG)*			
Huntington’s disease	165	0.024 (0.061)	0.353
Parkinson’s disease	108	−0.053 (0.074)	0.763

*ADHD* attention-deficit/hyperactivity disorder, *IR-MPH* immediate-release methylphenidate, *SNAP-IV* Swanson, Nolan, and Pelham Rating Scale, version 4, *SE* standard

^a^FDR = 0.117

^b^FDR = 0.117

## Discussion

This study investigated the global protein alterations induced by MPH in the cortex of rats. The 98 DEPs identified were analyzed in the context of biological pathways to provide a comprehensive overview of which systems are affected by MPH administration. The overall findings pointed out some pathways presenting biological plausibility, such as those related to synaptic neurotransmission, and others with a less established relationship that should be further explored, such as those involving oxidative stress, respiratory chain, and metabolic processes. The major relevance of this exploratory approach, besides unraveling putative molecular effects of MPH, is the perspective to translate the results to clinical samples. In this sense, we were also able to reinforce the relevance of some findings in a clinical sample of adults with ADHD, for which we found suggestive evidence on the association between MPH treatment response and genetic variability in pathways related to neurotransmitter release and GABA transmission.

Among the most interesting pathways overrepresented in the functional enrichment analysis of the DEPs with MPH treatment are those involved in synaptic neurotransmission, including gene-sets related to neurotransmitter release and transport, vesicle-mediated transport and GABA transmission (see Fig. 2 and Supplementary Tables 2 and 3). In line with these results, it has been reported that MPH induces alterations in synaptic vesicle-mediated neurotransmission in lysates of striatal synaptosomes of rats, where the content, transport, and release of dopamine were altered with MPH treatment^42,43^. Additionally, gene expression levels of proteins involved in neurotransmitter release were reduced with MPH treatment in PC12 cells^10^, and an enrichment analysis from transcriptomics studies also indicated the involvement of the synaptic transmission pathway in the MPH treatment^15^. Regarding the GABA neurotransmitter specifically, there are also evidences that MPH can modulate the GABAergic transmission^44,45^.

The involvement of pathways related to synaptic neurotransmission in MPH treatment response was reinforced by the gene-set analysis using a clinical sample of adults with ADHD, which was based on the biological pathways generated from the functional enrichment of the DEPs in WKY rats. We adopted this strategy considering that pathways modulated by MPH at the proteomic level are a good source of biological candidates to be investigated regarding treatment response. This translational approach suggests that among the pathways altered by MPH in rats, those involved in neurotransmitter release and GABA transmission are also important for treatment response to MPH. Although similar findings were observed only in men in the analysis separated by sex, this does not suggest an apparent sex-specific effect since the stratification did not improve the association values, indicating that the inclusion of women in the analysis may contribute for the association as well. Therefore, genetic variants in such biological pathways could be an important source of treatment response variability. Indeed, SNARE-related genetic variants, involved in neurotransmitter exocytosis, have been associated with treatment response to MPH in candidate gene association studies, in particular, *SNAP-25* in children^46^ and *SYT1* in adults^39^.

In line with this, a review of proteomic studies on multiple addictive drugs reported that synaptic proteins (comprising those related to the SNARE complex and the GABA receptor signaling) were also associated with exposure to one or more addictive drugs, including cocaine and/or amphetamine/methamphetamine^47^, suggesting an important role of these proteins for the effects of psychostimulants. Besides these proteins, most pathways modulated by MPH in our study overlap with those previously reported for other psychostimulants, such as alterations in metabolism, oxidative stress, and cell signaling^26,47^. On the other hand, the overrepresentation of pathways related to cardiac processes in our study is not shared with proteomic results for other psychostimulants^26,47^. Although MPH-induced alterations in the cardiovascular system, e.g., heart rate, is reasonable since they have been reported in patients with ADHD^48^, it is also possible that proteins comprising these pathways are indeed more related to other biological systems in this context. For example, a group of genes included in “regulation of cardiac conduction” overlaps with “muscle contraction”, which are more specific gene-sets, but they also comprise pathways involving cell communication by electrical coupling, calcium ion signaling, and ion homeostasis, which are very general signaling processes likely relevant for several systems, and more plausible for MPH effects in the brain (see Fig. 2 and Supplementary Tables 2 and 3).

Transcriptomics studies also suggest effects of MPH on synaptic transmission and cell signaling related pathways;^15^ however, they also point out other pathways, including axon guidance, nervous system development, Wnt signaling, cell/neuron differentiation, neuropeptide signaling, which were not observed in our study. Even though our methods differ from those used in these studies, previous investigations presenting comparable experimental designs also report a low concordance between proteomic and transcriptomic techniques. Such limited correlation between mRNA levels and protein abundance is a result of complex post-transcriptional and -translational regulatory mechanisms, such as transcript stability and protein degradation, that are reflected in the intrinsic differences captured in each step of the flow of genetic information^49^. Ideally, they should be interpreted as complementary approaches, but since proteomics detects the final product of genes, it can provide more direct insights on functional mechanisms and cellular activities.

Besides, the enrichment analysis of the DEPs in our study also retrieved interesting results from the KEGG category. Among them, the Huntington and Parkinson gene-sets might indicate an involvement of genes related to movement and cognitive processes in MPH actions. This might suggest that genetic variation related to traits underlying these diseases could be linking such pathways with MPH effects. Interestingly, the review mentioned above of proteomic studies involving the exposure to addictive drugs also pointed out Huntington’s disease signaling pathway^47^.

The functional enrichment results should be interpreted considering the limitation of this approach regarding the possibility of false-positive results in hypergeometric-based tests, which is a consequence of the gene overlap among the gene-sets (as exemplified above for the “regulation of cardiac conduction” gene-set). However, there is a high concordance among the pathways generated by the three tools used for analysis. Also, the associations observed for the clinical sample of adults with ADHD treated with MPH extended some of the proteomic findings from rats, at least in terms of treatment response. Unfortunately, we were not able to assess other system effects in the clinical sample, such as the cardiovascular and oxidative stress processes that have been reported for patients treated with MPH. Moreover, the sample size for the clinical sample is relatively small, and this could be limiting the detection of additional associations. Among the experimental limitations, we should mention we used WKY male rats, and the results might not be generalized to females. Also, we did not consider the behavioral effects of MPH treatment, and our results are reflecting MPH-induced molecular modifications independently of the presence of ADHD symptoms. Therefore, our results should be interpreted similarly to basic research and nonclinical drug development studies, for which the effects of a medication are evaluated without biological interference related to the disease at the first moment. For further interpretation of our results in the context of ADHD, they should be replicated using animal models for ADHD. The exclusive proteins of the saline-treated group (DDHD1, TNRC6B, ATXN1, and PRODH1) may raise the possibility of an artifact. However, since these proteins were consistently detected among the biological replicates in this condition, a possible suppressing effect of MPH in their expression is more likely. Although the mechanisms underlying these putative effects remain to be elucidated, their relevance in neurodevelopmental phenotypes has already been demonstrated^50–57^. Finally, considering the exploratory nature of our study design, cortex was the only brain region evaluated, and we did not perform methodological replication; however, the enrichment analysis and the extension of some results to a clinical sample allow us to make inferences on the most important biological pathways and candidates that should be further explored in future studies and in different brain regions.

In general, our exploratory study unraveled several pathways from different biological processes that are modulated by MPH treatment in rats. Importantly, the findings involving processes related to synaptic neurotransmission were extended to humans for MPH treatment response. The overall evidence from previous experimental studies combined with our findings suggests that MPH response involves a complex interaction of neurotransmitter systems that go beyond the widely acknowledged dopamine and norepinephrine, especially related to GABA transmission. This translational perspective, that integrates proteomics and pharmacogenomics, can shed light on the molecular signatures of MPH and the possible biological sources of treatment response variability.

## Supplementary information

Supplementary Material
