# Supplementary material for: Integrative proteomics and pharmacogenomics analysis of methylphenidate treatment response
Source: Transl Psychiatry. 2019 Nov 18;9:308. doi: 10.1038/s41398-019-0649-5 (PMC6861257; doi:10.1038/s41398-019-0649-5)
Supplement: Supplementary file 1 — Supplementary Material [file 41398_2019_649_MOESM1_ESM.doc]

**Supplemental Information**

**Supplemental Methods**

*Sample preparation and protein extraction for mass spectrometry*

The cortex tissues were individually homogenized and sonicated on cold HEPES 50mM/sucrose 0.25M buffer (pH 7.5) containing 1% Triton X-100 (Sigma), 1% halt EDTA-free protease inhibitor (ThermoFisher Scientific), 1% phosphatase inhibitor cocktail A (Santa Cruz Biotechnologies), 1% EDTA, 30mM sodium fluoride and 1mM sodium orthovanadate. For protein extraction, methanol, chloroform, and water were added to the tissue homogenates, and after mixing and centrifuging at 13 000 x g for 1 min, the upper layer was removed. Methanol was added to the remaining solution followed by another centrifugation step at 13 000 x g for 2 min. The supernatant was discarded and the remaining final pellet was stored at -80ºC until further processing.

Protein extracts were resuspended with 8M urea 50mM ammonium bicarbonate and protein concentration was determined using a bicinchoninic acid (BCA) protein assay (Thermo Scientific). Disulfide bonds were reduced with 5 mM tris (2-carboxyethyl) phosphine (TCEP) at 30 °C for 60 min and cysteines were subsequently alkylated (carbamidomethylated) with 15 mM iodoacetamide (IAA) in the dark at room temperature for 30 min. Urea was then diluted to 1 M urea using 50 mM ammonium bicarbonate to a final volume of 500 uL, and proteins were subjected to overnight digestion with mass spec grade Trypsin/Lys-C mix (Promega). After digestion, proteins were acidified with formic acid (FA) and subsequently desalted using AssayMap C18 cartridges (Agilent) mounted on an Agilent AssayMap BRAVO liquid handling system. C18 cartridges were first conditioned with 100% acetonitrile (ACN), followed 0.1% FA. The samples were then loaded onto the conditioned C18 cartridge, washed with 0.1% FA, and eluted with 60% ACN, 0.1% FA. Finally, the organic solvent was removed in a SpeedVac concentrator prior to LC-MS/MS analysis.

*Data analysis*

Using MaxQuant, precursor mass tolerance was set to 20 ppm for the first search where initial mass recalibration was completed and 4.5 ppm for the main search. Product ions were searched with a mass tolerance of 0.5 Da. The maximum precursor ion charge state used for searching was 7. Carbamidomethylation of cysteines was searched as a fixed modification, while oxidation of methionines and acetylation of protein N-terminal were searched as variable modifications. Enzyme was set to trypsin in a specific mode and a maximum of two missed cleavages was allowed for searching. The target-decoy-based false discovery rate (FDR) filter for spectrum and protein identification was set to 1%.

For label-free protein quantitative analysis, peptide intensities were log base 2 transformed and normalized. A total of 4 normalization strategies were deployed using either *loess* or *vsn* (Variance Stabilization and Normalization) algorithms within each condition or across all samples (global). Machine learning was used to determine the optimal normalization method, herein global *loess* normalization, for subsequent analysis using MSstats bioconductor package and determination of the differentially expressed proteins.

**Supplementary Table 1.** Characteristics of the clinical sample of patients with ADHD that underwent IR-MPH treatment for at least 30 days.

| Characteristic | n = 189 |
| --- | --- |
|  | *n (%)* |
| Sex (men) | 102 (54.0) |
| Lifetime comorbidities |  |
| Tobacco use disorder | 82 (43.4) |
| Oppositional defiant disorder | 124 (65.6) |
| Antisocial personality disorder | 15 (7.9) |
| Substance use disorder | 33 (17.5) |
| Bipolar disorder | 31 (16.4) |
| Major depressive disorder | 71 (37.6) |
| Generalized anxiety disorder | 41 (21.7) |
| Phobia^a^ | 47 (24.9) |
| Panic disorder | 8 (4.2) |
| Use of concomitant psychiatric medications^b^ | 62 (32.8) |
|  | *Mean (s.d.)* |
| Age | 33.58 (10.7) |
| IR-MPH daily dose prescribed (mg/kg) | 0.54 (0.2) |
| Baseline CGI-S scores | 4.61 (0.73) |
| Baseline SNAP-IV scores of ADHD symptoms | 1.74 (0.52) |
| Percentage change in ADHD symptoms after IR-MPH treatment | -52.8 (26.1) |

Abbreviations: ADHD, attention-deficit/hyperactivity disorder; CGI-S, clinical global impression – severity; IR-MPH, immediate-release methylphenidate; *s.d.*, standard deviation; SNAP-IV, Swanson, Nolan, and Pelham Rating Scale, version 4. ^a^ Includes specific and social phobias. ^b^ The most frequent psychiatric medications includes antidepressants (69.3%) and mood stabilizers (19.4%).

**Supplementary Table 2.** DAVID results of the overrepresented gene-sets retrieved from the functional enrichment analysis of the 97 differentially expressed proteins with MPH treatment in the cortex of Wistar-Kyoto rats.

| **Gene-set** | **Genes^a^** | **P-value** | **FDR** |
| --- | --- | --- | --- |
| **Gene-Ontology (GO) - Biological process** |  |  |  |
| Vesicle-mediated transport (GO:0016192) | Arf6, Sec22b, Copg2, Gosr2, Sar1a | 0.0005 | 0.2516 |
| Transmembrane transport (GO:0055085) | Aqp4, Mcu, Slc25a4, Slc6a1, Slc6a11, Sv2a, Sv2b | 0.0009 | 0.2492 |
| Learning (GO:0007612) | Atp8a1, Grin2b, Slc6a1, Slc8a2 (4.1%) | 0.0047 | 0.6175 |
| Telencephalon development (GO:0021537) | Nrgn, Slc8a1, Slc8a2 | 0.0131 | 0.8656 |
| Neurotransmitter transport (GO:0006836) | Slc6a1, Slc6a11, Sv2a | 0.0131 | 0.8656 |
| Translation (GO:0006412) | Eif5b, Mrps5, Rpl19, Rps5, Rpl38, Slc25a10, Slc25a4 | 0.0132 | 0.8001 |
| Cellular calcium ion homeostasis (GO:0006874) | Atp2b1, Slc8a1, Slc8a2, Sv2a | 0.0136 | 0.7504 |
| Calcium ion transmembrane transport (GO:0070588) | Atp2b1, Gpm6a, Slc8a1, Slc8a2 | 0.0195 | 0.8184 |
| Calcium ion export (GO:1901660) | Atp2b1, Slc8a1 | 0.0204 | 0.7903 |
| Regulation of vascular endothelial growth factor production (GO:0010574) | Ndrg2, Aqp4 | 0.0254 | 0.8237 |
| Neurotransmitter uptake (GO:0001504) | Sv2a, Sv2b | 0.0254 | 0.8237 |
| Protein transport (GO:0015031) | Arf6, Sec22b, Cadps, Hap1, Jakmip1 | 0.0433 | 0.9320 |
| **Reactome** |  |  |  |
| Muscle contraction (R-RNO-5578775) | Atp1a1, Atp2b1, Prkaca, Slc8a1, Slc8a2 | 0.0003 | 0.0389 |
| Hemostasis (R-RNO-418359) | Atp2b1, Slc8a1, Slc8a2 | 0.0034 | 0.1872 |
| Metabolism of proteins/  Vesicle-mediated transport (R-RNO-5694530) | Sec22b, Gosr2, Tmed10 | 0.0151 | 0.4638 |
| Neuronal System (R-RNO-888593) | Slc6a1, Slc6a11 | 0.0250 | 0.5402 |
| Transport of small molecules (R-RNO-936837) | Atp1a1, Atp8a1, Atp2b1 | 0.0364 | 0.5986 |
| **Kyoto Encyclopedia of Genes and Genomes (KEGG)** |  |  |  |
| Huntington's disease (rno05016) | Ndufs2, Ndufa1, Ndufb6, Cltc, Cox6a1, Grin2b, Hap1, Slc25a4 | 0.0004 | 0.0698 |
| GABAergic synapse (rno04727) | Gabarapl1, Gad1, Hap1, Prkaca, Slc6a1 | 0.0031 | 0.2076 |
| Protein digestion and absorption (rno04974) | Atp1a1, Col4a1, Prss1, Slc8a1, Slc8a2 | 0.0033 | 0.1550 |
| Parkinson's disease (rno05012) | Ndufs2, Ndufa1, Ndufb6, Cox6a1, Prkaca, Slc25a4 | 0.0040 | 0.1402 |
| cGMP-PKG signaling pathway (rno04022) | Atp1a1, Atp2b1, Gtf2i, Slc25a4, Slc8a1, Slc8a2 | 0.0052 | 0.1465 |
| Bile secretion (rno04976) | Atp1a1, Aqp4, Nceh1, Prkaca | 0.0131 | 0.2839 |
| Alzheimer's disease (rno05010) | Ndufs2, Ndufa1, Ndufb6, Cox6a1, Grin2b | 0.0375 | 0.5635 |
| Calcium signaling pathway (rno04020) | Atp2b1, Prkaca, Slc25a4, Slc8a1, Slc8a2 | 0.0394 | 0.5342 |
| Endocrine and other factor-regulated calcium reabsorption (rno04961) | Atp1a1, Cltc, Prkaca | 0.0422 | 0.5172 |

^a^Inputted genes that are part of the enriched gene-sets.

**Supplementary Table 3.** Enrichr results of the overrepresented gene-sets retrieved from the functional enrichment analysis of the 97 differentially expressed proteins with MPH treatment in the cortex of Wistar-Kyoto rats.

| **Gene-set** | **Genes^a^** | **P-value** | **FDR** |
| --- | --- | --- | --- |
| **Gene-Ontology (GO) - Biological process** |  |  |  |
| Regulation of cardiac conduction (GO:1903779) | Atp1a1, Atp2b1, Prkaca, Slc8a1, Slc8a2 | <0.0001 | 0.1062 |
| Cell communication by electrical coupling involved in cardiac conduction (GO:0086064) | Atp1a1, Prkaca, Slc8a1 | <0.0001 | 0.1019 |
| Regulation of heart contraction (GO:0008016) | Atp1a1, Atp2b1, Prkaca, Slc8a1, Slc8a2 | 0.0001 | 0.1863 |
| Protein complex assembly (GO:0006461) | Pfkl, Tmed10, Gosr2, Sec22b, Rbmx, Mcu | 0.0003 | 0.4421 |
| Vesicle fusion with Golgi apparatus (GO:0048280) | Gosr2, Sec22b | 0.0004 | 0.3592 |
| COPII-coated vesicle budding (GO:0090114) | Tmed10, Sar1a, Gosr2, Sec22b | 0.0004 | 0.3122 |
| Ion transport (GO:0006811) | Atp8a1, Aqp4, Slc25a10, Atp1a1, Atp2b1, Slc8a1, Slc8a2 | 0.0006 | 0.4104 |
| Endoplasmic reticulum organization (GO:0007029) | Reep1, Atl1, Lman2 | 0.0007 | 0.4250 |
| Retrograde vesicle-mediated transport, Golgi to ER (GO:0006890) | Tmed10, Lman2, Copg2, Sec22b | 0.0007 | 0.3830 |
| Central nervous system development (GO:0007417) | Atxn1, Mog, Col4a1, Elp3, Hap1, Grin2b | 0.0007 | 0.3653 |
| ATP synthesis coupled electron transport (GO:0042773) | Ndufb6, Ndufs2 | 0.0008 | 0.3881 |
| Golgi vesicle budding (GO:0048194) | Tmed10, Atp8a1 | 0.0008 | 0.3558 |
| Mitochondrial ATP synthesis coupled electron transport (GO:0042775) | Ndufb6, Ndufa1, Ndufs2, Cox6a1 | 0.0008 | 0.3322 |
| Calcium ion transmembrane transport (GO:0070588) | Grin2b, Slc8a1, Mcu, Slc8a2 | 0.0009 | 0.3221 |
| Translation (GO:0006412) | Rps5, Tars, Rpl38, Tars2, Mrps5, Rpl19 | 0.0010 | 0.3439 |
| **Reactome** |  |  |  |
| Ion homeostasis (R-HSA-5578775) | Atp1a1, Atp2b1, Prkaca, Slc8a1, Slc8a2 | <0.0001 | 0.0077 |
| Cargo concentration in the ER (R-HSA-5694530) | Tmed10, Gosr2, Lman2, Sec22b | <0.0001 | 0.0152 |
| Reduction of cytosolic Ca levels (R-HSA-418359) | Atp2b1, Slc8a1, Slc8a2 | <0.0001 | 0.0124 |
| Metabolism (R-HSA-1430728) | Cox19, Prss1, Ndufb6, Rps5, Mgst3, Ndufa1, Slc6a11, Ddhd1, Cox6a1, Fdx1l, Gys1, Por, Pfkl, Nceh1, Ogdh, Ndufs2, Rpl38, Slc25a10, Prkaca, Prodh, Acss1, Slc25a4, Rpl19 | <0.0001 | 0.0144 |
| Metabolism of proteins (R-HSA-392499) | Cox19, Eif5b, Tmed10, Rpn2, Ssr4, Rps5, Gosr2, Csnk2a2, Smc3, Lman2, Uba2, Rpl38, Copg3, Sec22b, Slc25a4, Rpl19 | 0.0001 | 0.0194 |
| Respiratory electron transport (R-HSA-611105) | Cox19 , Ndufb6 , Ndufa1 , Ndufs2 , Cox6a1 | 0.0001 | 0.0185 |
| Translation (R-HSA-72766) | Eif5b , Rpn2 , Ssr4 , Rps5 , Rpl38 , Rpl19 | 0.0001 | 0.0218 |
| GABA synthesis, release, reuptake and degradation (R-HSA-888590) | Gad1 , Slc6a11 , Slc6a1 | 0.0001 | 0.0200 |
| The citric acid (TCA) cycle and respiratory electron transport (R-HSA-1428517) | Cox19 , Ndufb6 , Ogdh , Ndufa1 , Ndufs2 , Cox6a1 | 0.0001 | 0.0182 |
| SRP-dependent cotranslational protein targeting to membrane (R-HSA-1799339) | Rpn2 , Ssr4 , Rps5 , Rpl38 , Rpl19 | 0.0002 | 0.0279 |
| Respiratory electron transport, ATP synthesis by chemiosmotic coupling, and heat production by uncoupling proteins. (R-HSA-163200) | Cox19 , Ndufb6 , Ndufa1 , Ndufs2 , Cox6a1 | 0.0002 | 0.0277 |
| Toxicity of botulinum toxin type D (BoNT/D) (R-HSA-5250955) | Sv2b , Sv2a | 0.0002 | 0.0300 |
| Toxicity of botulinum toxin type F (BoNT/F) (R-HSA-5250981 | Sv2b , Sv2a | 0.0002 | 0.0277 |
| Platelet calcium homeostasis (R-HSA-418360) | Atp2b1 , Slc8a1 , Slc8a2 | 0.0003 | 0.0335 |
| COPII (Coat Protein 2) Mediated Vesicle Transport (R-HSA-204005) | Tmed10 , Gosr2 , Lman2 , Sec22b | 0.0003 | 0.0354 |
| ER to Golgi Anterograde Transport (R-HSA-199977) | Tmed10 , Gosr2 , Lman2 , Copg2 , Sec22b | 0.0005 | 0.0445 |
| Cardiac conduction (R-HSA-5576891) | Atp1a1 , Atp2b1 , Prkaca , Slc8a1 , Slc8a2 | 0.0005 | 0.0481 |
| **Kyoto Encyclopedia of Genes and Genomes (KEGG)** |  |  |  |
| Huntington disease | Ndufb6, Cltc, Ndufa1, Ndufs2, Hap1, Cox6a1, Grin2b, Slc25a4 | <0.0001 | 0.0014 |
| GABAergic synapse | Gabarapl1, Gad1, Slc6a11, Hap1, Slc6a1, Prkaca | <0.0001 | 0.0008 |
| Endocrine and other factor-regulated calcium reabsorption | Cltc, Atp1a1, Atp2b1, Prkaca, Slc8a1 | <0.0001 | 0.0007 |
| Parkinson disease | Ndufb6, Ndufa1, Ndufs2, Cox6a1, prkaca, slc25a4 | 0.0001 | 0.0058 |
| cGMP-PKG signaling pathway | Atp1a1, Atp2b1, Slc25a4, Slc8a1, Slc8a2, Gtf2i | 0.0002 | 0.0123 |
| Calcium signaling pathway | Atp2b1, Prkaca, Slc25a4, Slc8a1, Mcu, Slc8a2 | 0.0003 | 0.0170 |
| Bile secretion | Nceh1, Aqp4, Atp1a1, Prkaca | 0.0004 | 0.0187 |
| Thermogenesis | Cox19, Ndufb6, Ndufa1, Ndufs2, Cox6a1, Prkaca | 0.0010 | 0.0366 |
| Protein digestion and absorption | Col4a1, Atp1a1, Slc8a1, Slc8a2 | 0.0010 | 0.0338 |
| Mineral absorption | Atp1a1, Atp2b1, Slc8a1 | 0.0013 | 0.0396 |
| Alzheimer disease | Ndufb6, Ndufa1, Ndufs2, Cox6a1, Grin2b | 0.0017 | 0.0470 |

^a^Inputted genes that are part of the enriched gene-sets.

**Supplementary Table 4.** Competitive gene-set analysis of the percentage change in symptoms of ADHD according to SNAP-IV scale after treatment with IR-MPH in adults with ADHD stratified by sex.

| **Gene-set** |  | **Nº genes** |  | **Women** | |  | **Men** | |
| --- | --- | --- | --- | --- | --- | --- | --- | --- |
|  |  |  |  | **Beta (SE)** | **P-value** |  | **Beta (SE)** | **P-value** |
| **Gene-Ontology (GO) - Biological process** |  |  |  |  |  |  |  |  |
| Organonitrogen compound metabolic process |  | 1685 |  | 0.027 (0.020) | 0.092 |  | -0.005 (0.021) | 0.598 |
| Generation of precursor metabolites and energy |  | 272 |  | -0.031 (0.048) | 0.743 |  | -0.017 (0.049) | 0.635 |
| Oxidation reduction process |  | 840 |  | 0.008 (0.028) | 0.391 |  | 0.006 (0.030) | 0.418 |
| Cellular respiration |  | 134 |  | -0.053 (0.068) | 0.783 |  | 0.020 (0.067) | 0.385 |
| Energy derivation by oxidation of organic compounds |  | 202 |  | -0.023 (0.056) | 0.662 |  | -0.029 (0.057) | 0.699 |
| Regulation of cardiac conduction |  | 63 |  | -0.073 (0.102) | 0.762 |  | -0.230 (0.107) | 0.984 |
| Cell communication by electrical coupling |  | 15 |  | -0.318 (0.237) | 0.910 |  | -0.001 (0.259) | 0.502 |
| **Reactome** |  |  |  |  |  |  |  |  |
| GABA synthesis release reuptake and degradation |  | 17 |  | 0.165 (0.204) | 0.209 |  | 0.360 (0.209) | **0.043^a^** |
| Translation |  | 133 |  | 0.031 (0.067) | 0.323 |  | -0.002 (0.069) | 0.502 |
| SRP dependent cotranslational protein targeting to membrane |  | 100 |  | 0.073 (0.078) | 0.174 |  | -0.057 (0.079) | 0.766 |
| Transmembrane transport of small molecules |  | 391 |  | 0.009 (0.041) | 0.414 |  | 0.006 (0.044) | 0.447 |
| TCA cycle and respiratory electron transport |  | 111 |  | -0.138 (0.072) | 0.973 |  | -0.010 (0.073) | 0.556 |
| Respiratory electron transport |  | 62 |  | -0.112 (0.095) | 0.880 |  | 0.069 (0.096) | 0.234 |
| Glucose metabolism |  | 59 |  | -0.231 (0.094) | 0.993 |  | 0.117 (0.097) | 0.115 |
| Neurotransmitter release cycle |  | 32 |  | 0.171 (0.149) | 0.126 |  | 0.366 (0.155) | **0.009^b^** |
| Respiratory electron transport ATP synthesis by chemiosmotic coupling and heat production by uncoupling proteins |  | 78 |  | -0.168 (0.085) | 0.976 |  | -0.028 (0.085) | 0.627 |
| **Kyoto Encyclopedia of Genes and Genomes (KEGG)** |  |  |  |  |  |  |  |  |
| Huntington’s disease |  | 165 |  | -0.089 (0.061) | 0.928 |  | -0.036 (0.062) | 0.717 |
| Parkinson’s disease |  | 108 |  | -0.153 (0.075) | 0.979 |  | -0.042 (0.076) | 0.710 |

^a^FDR = 0.387; ^b^FDR = 0.162. Abbreviations: ADHD, attention-deficit/hyperactivity disorder; IR-MPH, immediate-release methylphenidate; SNAP-IV, Swanson, Nolan, and Pelham Rating Scale, version 4; SE, standard error.


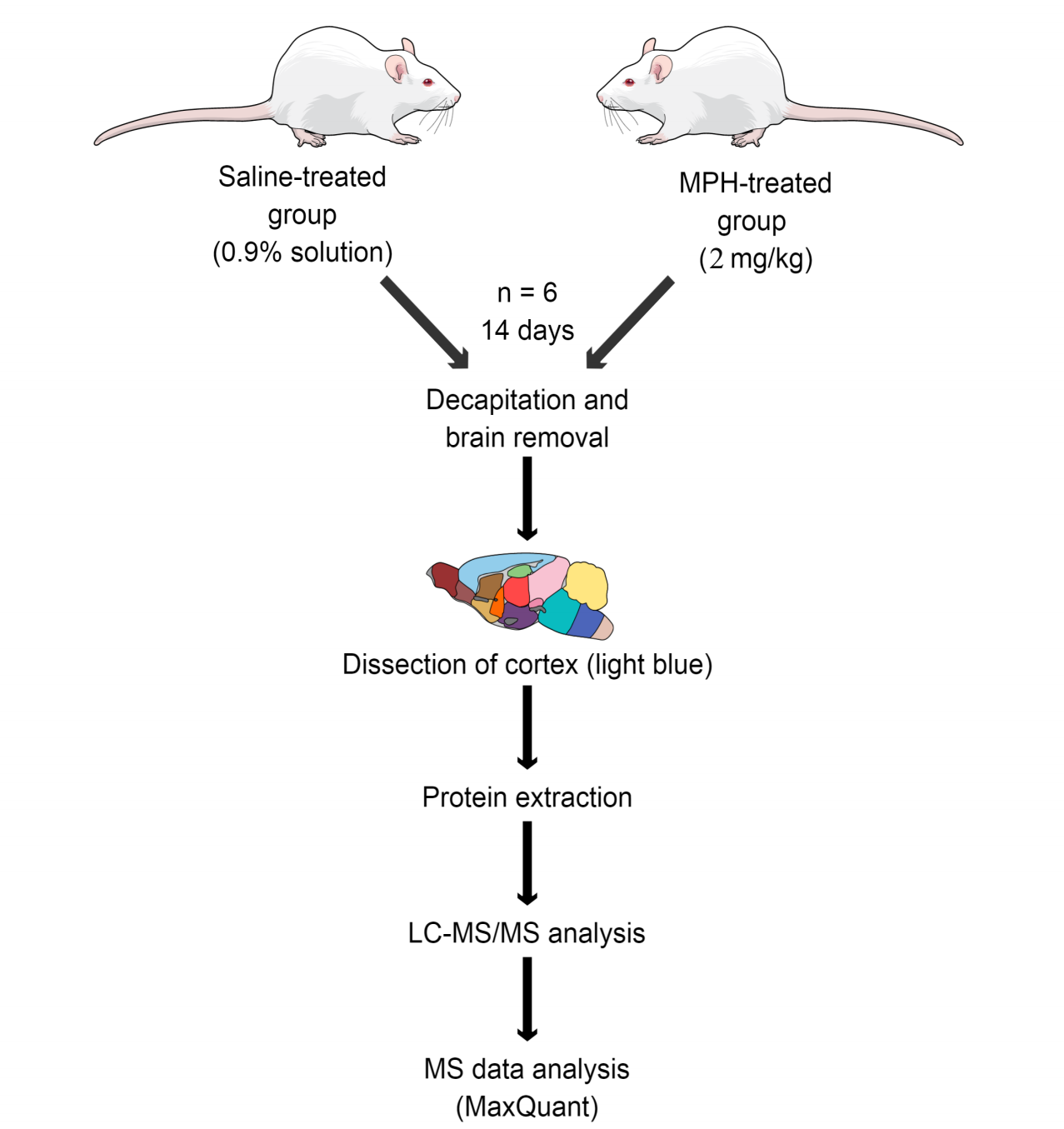


**Supplementary Figure 1.** Flowchart of the experimental design of the study. Figure created in the Mind the Graph platform.
